# Osteosarcoma: Evolution of Treatment Paradigms

**DOI:** 10.1155/2013/203531

**Published:** 2013-05-27

**Authors:** Norman Jaffe, Ajay Puri, Hans Gelderblom

**Affiliations:** ^1^Children's Cancer Hospital, University of Texas M.D Anderson Cancer Center, Houston, TX 77030, USA; ^2^Department of Surgical Oncology, Tata Memorial Hospital, Mumbai, India; ^3^Department of Clinical Oncology, Leiden University Medical Center, Albinusdreef 2, 2300RC Leiden, The Netherlands

## Abstract

This paper reviews the contribution of chemotherapy in the conquest of osteosarcoma. It discusses how the treatment of osteosarcoma has evolved over the last five decades, resulting in a more than fivefold increase in survival. Though the initial improvements in survival were dramatic, essentially there has been no change in the outlook for this disease over the past 30 years. The paper also highlights the necessity of a multidisciplinary approach to combat this disease and stresses the need to explore newer treatment agents in order to build on the lessons learnt from the past while striving to achieve greater levels of success.

## 1. Introduction 

Bone cancers are rare in humans. In 2009, it is estimated that 2570 new cases of bone sarcomas were diagnosed in the United States [1]. Osteosarcoma is the most common. The term “osteosarcoma” as opposed to “osteogenic sarcoma” is preferred by the World Health Organization (WHO). The eponym was introduced by Boyer in 1805 [2]. In 1879, Gross published a paper entitled “Sarcoma of the Long Bone Based upon a Study of One Hundred and Sixty-five Cases” [3]. Most, if not all the tumors, were probably osteosarcoma. He advocated treatment by early amputation. The outcome was dismal; nonetheless it was accepted as the “standard” of treatment. 

In the course of the ensuing one and a half century, osteosarcoma became established as a distinct pathological and radiological entity with no change in the “standard” of therapy. The survival rate was less than 10%; in rare publications, it occasionally rose to 20%. The dismal survival was due to the biological behavior of the malignancy: pulmonary micrometastases were present in at least 80% of patients at diagnosis. These metastases were not visible on conventional imaging studies. However, they surfaced 8–12 months after amputation and were responsible for the patient's demise within 12 to 24 months of their appearance. Osteosarcoma therefore had to be considered a systemic disease with systemic therapy required for cure. Until the mid-20th century, no such therapy was available. 

## 2. Radiation Therapy 

In view of the poor prognosis with primary surgical ablation, Sir Stanford Cade a British Surgeon Radiotherapist in 1931 advocated radiation therapy to treat the primary tumor [4]. Following completion of therapy (6000 rad over six weeks) the patient was observed for the possible emergence of pulmonary metastases for 6–9 months; if metastases failed to appear, an elective amputation was performed. The intent was to avoid “futile mutilation” in a patient destined to die. It was also postulated that, in some patients, effective radiation with optimum local control might also avert amputation. A similar approach was employed by Ferguson at the Sloan Kettering Memorial Cancer Center in New York [5]. The strategy failed to meet its objectives. Tumor dissemination from a nonamputated limb remained a constant threat and failure of local control produced severe pain and protracted morbidity eventually requiring amputation for palliation in most patients. Cade in summarizing the prevailing treatment at a meeting for osteosarcoma concluded “Gentlemen if you operate they die, if you do not operate they die just the same; this meeting should be concluded with prayers.” 

 Radiation therapy was also administered to the lungs by the Mayo Clinic [6]. There was little effect on the long-term survival. 

## 3. Immunotherapy

A glimmer of hope emerged from preliminary studies in immunotherapy advocated by Marcove et al. [7] and Neff and Enneking [8] as therapy for destruction of the systemic micrometastases; however long-term results were disappointing. Fudenberg presented the preliminary results with Transfer Factor but it did not gain wide acceptance [9]. Strander et al. published the initial results with interferon [10]. This biological agent was also utilized by Swedish investigators and appeared to hold some promise. It is currently a component of the EURAMOS investigative study (vide infra).

## 4. Chemotherapy 

The discovery of chemotherapeutic agents which were active in osteosarcoma was a milestone in attempts to find a cure. This occurred in the 1960s after a few disappointing experiences. Nitrogen mustard had been administered concurrently with radiation therapy for treatment of the primary tumor (Dana Farber Institute, formerly the Children's Cancer Foundation, (NJ unpublished data)); it failed to prevent the emergence of overt pulmonary metastases. Similarly, regimens utilizing combinations of Nitrogen Mustard, Mitomycin C and Vincristine yielded minimal responses and were abandoned [11] However, an early report by Pinkel indicated possible activity with oral cyclophosphamide [12]. 

### 4.1. Compadri Regimens

“Conpadri” is an acronym for the combination of cyclophosphamide, Oncovin, vincristine (Oncovin), doxorubicin (Adriamycin), and L-phenylalanine mustard. With the addition of methotrexate the acronym changed to “Compadri.” It was developed by Sutow in the early 1960s [13]. L-phenylalanine mustard was shown to have mild antitumor effects. Temporary regressions in 10%–43% of patients were reported [14]. It was therefore administered as adjuvant therapy to nonmetastatic patients after surgical ablation of the primary tumor. A disease-free survival of 14% was attained [15]. In 1969, the combination of vincristine, actinomycin D (Dactinomycin), and cyclophosphamide (VAC) was investigated as adjuvant therapy for rhabdomyosarcoma and also found to be effective in osteosarcoma [16]. It was administered in an intensive “pulse” schedule based upon the understanding that cyclophosphamide was more effective when utilized in this manner. Twelve osteosarcoma patients were treated yielding a 33% disease-free survival. This laid the cornerstone for the construction of the “Conpadri/Compadri” regimens. With the demonstration that doxorubicin was highly effective in osteosarcoma (see below), Sutow substituted doxorubicin for actinomycin D [13]. 

The Compadri regimens constituted the first rational attempt to employ combination chemotherapy for patients as adjuvant postoperative chemotherapy. They comprised agents with different mechanisms of action and minimal overlapping toxicity. Compadri I–III yielded a 41% 18 + month disease-free survival [17]. 

### 4.2. High Dose Methorexate

High dose methotrexate was a major weapon in the armamentarium of treatment for osteosarcoma. It demonstrated that the disease was indeed responsive to chemotherapy; it also ignited tremendous controversy. No other agent was subjected to similar criticism. It was the only drug among the effective agents which was subjected to a comparative trial of efficacy with another agent (cisplatin) [18].

Methotrexate was discovered by Farber et al. in the 1940s and was a pivotal agent for the cure of childhood leukemia and lymphoma [19]. It acts by depriving the cell of folates which is essential for the formation of DNA. The antidote is leucovorin which can reverse its activity and abort and treat toxicity. Except for osteosarcoma, there are no reports of its efficacy in childhood solid tumors. 

A novel strategy to increase the efficacy of methotrexate in leukemia was devised by Abraham Goldin. He administered large (toxic) doses of the drug to leukemia bearing mice and after a defined period “rescued” them with leucovorin [20]. Toxicity was aborted and cure was achieved. The regimen was investigated by Djerassi et al. in childhood lymphoma and leukemia and found to be safe and effective [21].

Farber held a weekly tumor board conference at the Dana Farber Cancer Institute. Djerassi was invited to present his data on methotrexate/leucovorin at one of the meetings; the presentation was novel and intriguing and well received. In view of the absence of any known effective chemotherapeutic agent in osteosarcoma, NJ requested permission from Farber to investigate the regimen in this disease. During this period (the 1960s), therapeutic research was in its infancy and Institutional Review Boards and Surveillance Committees had not been formally mandated or established. Permission to conduct investigations was generally obtained from senior investigators, consultants, or directors or was decided by consensus among attending physicians. Permission was granted by Farber for the regimen to be administered to a patient who had developed pulmonary metastases six months after a hemipelvectomy. The potential side effects were outlined and consent for treatment from the parent was obtained. Complete disappearance of the metastases was achieved! The result was published [22]. 

The scientific community was kept abreast of the efficacy and potential toxicity of high dose methotrexate through follow-up investigations: toxicity was low and acceptable although an occasional death was reported from renal failure, hepatic failure, or superimposed infection from myelosuppression [23, 24]. The incidence of toxicity was subsequently reduced by assays that measured serum methotrexate levels (permitting construction of a methotrexate decay curve) and improved expertise, familiarity with the drug regimen, accumulating knowledge in its administration, and methods to treat and abort toxicity [25]. 

Methotrexate with leucovorin was also found to be nonmyelosuppressive and could be combined safely with other agents. When administered preoperatively, generally in preparation for limb salvage, and postoperatively as adjuvant therapy, survival was escalated to 60%–75% [26]. In addition with multimodal intervention comprising the possible administration of alternate agents and surgical resection to remove local recurrence and persistent or recurrent metastases, survival was escalated by an additional 10%−15% [27]. The discovery of effective chemotherapy was instrumental in implementing aggressive surgical sustained attacks (principally thoracotomies) to ablate recurrent and persistent tumor.

A major point of contention to the introduction and use of methotrexate was the report that the improved survival alleged to have occurred with methotrexate had been derived by comparison with survival in historical controls as opposed to concurrent controls [28–30]. The argument was bolstered by a concurrent control trial by the Mayo Clinic comparing methotrexate and leucovorin and amputation versus amputation only. There was apparently no improvement from the administration of methotrexate [31]. The above criticism was addressed by demonstrating that there had been no change in survival in several publications over the past half century, principally in reports published in the 1960s and 1970s [32, 33]. Eventually a two-arm randomized trial, MIOS, was launched utilizing concurrent controls: surgical ablation and chemotherapy were employed in one arm and surgical ablation only in the other “control” arm. Chemotherapy comprised methotrexate in combination with other agents [34]. Surgical ablation and combination chemotherapy therapy yielded a 65% survival whereas survival in the control arm (surgery only) was superimposable on historical controls, 5%! The result was repeated in a second similar, almost parallel running, trial conducted by Eilber et al. [35]. The outcome of the MIOS investigation and the contentious nature of the prevailing atmosphere were addressed by NJ in a letter to the New England Journal of Medicine [36] and the Eilber study in an editorial by Holland in the Journal of Clinical Oncology [37]. Numerous publications followed attesting to the efficacy of chemotherapy in osteosarcoma.

Forty years after the appearance of the first report of treatment with high dose methotrexate with leucovorum rescue in osteosarcoma an editorial in the Journal of Clinical Oncology reiterated the efficacy of the agent [38]. Among the references cited was a publication that *“non-methotrexate based therapy was a major poor prognostic factor”* for survival (NJ author emphasis) [39]. Of note also was a separate publication that three patients were cured without surgical resection of the primary tumor. They were treated with chemotherapy comprising high dose methotrexate, doxorubicin, and cisplatin. One patient achieved an initial response exclusively with methotrexate [40].

### 4.3. Doxorubicin

Doxorubicin was shown to be active in osteosarcoma in the 1960s [41, 42]. It constitutes the major component of the Compadri and other regimens utilized in osteosarcoma [17, 43, 44]. It acts by intercalating into DNA and inducing topoisomerase II-mediated single- and double-strand breaks. When administered alone or in combination with decarbazine and other agents it produced responses in 30%–40% of patients with a variety of cancers including patients with pulmonary metastases. It also potentiates the action of radiation therapy. Extravasation of the drug may cause ulceration. However its major toxic effect is cardiac failure; the total cumulative dose is generally limited to 300 mg/m^2^ in children under 6 years and 450 mg/m^2^ in adults. It is employed as combination therapy in pre- and postoperative regimens. 

### 4.4. Cisplatin

Cisplatin was first used in the treatment of osteosarcoma in the 1970s. It exerts its cytotoxic effect by platination of DNA. It has been administered by the intravenous and intra-arterial routes. Intravenously it produced a 30%–60% response in patients with metastatic disease. The response rate via the intra-arterial route is 60%–90% [45–48]. The intra-arterial route was introduced in an attempt to enhance the efficacy of therapy when it was surmised that alternate modes of therapy would possibly be helpful in advancing treatment of the disease. This route achieves higher local cytotoxic and concurrently effective systemic concentrations [48]. The angiogram utilized for intra-arterial administration was useful for assessing response by its effect on tumor neovascularity and stain. Unfortunately the intra-arterial route is labor intensive and generally requires conscious sedation or general anesthesia. Its use is therefore generally limited to selective circumstances. It was considered extremely useful in treating pathological fractures and in assessing a *rapid *response and the efficacy of treatment. Similar responses were achieved with intravenous cisplatin in combination with other agents and hence this approach has generally replaced the administration of intra-arterial cisplatin.

### 4.5. Oxazaphosphorines

Cyclophosphamide and ifosfamide were the two major alkylating agents used in the treatment of osteosarcoma. They require hepatic microsomes for activation. They were often used in combination with etoposide. The discovery of MESNA to prevent hemorrhagic cystitis permitted their administration in high doses. Response rates of 10%–40% have been reported [49–51]. The response rates can often be escalated by increasing the dose. The agents are not cross-resistant and therefore not mutually exclusive; responses may be achieved with the alternate agent if relapse has occurred with one agent. The drugs are used in preoperative and postoperative regimens, generally in combination with other agents.

## 5. Chemotherapy and Biological Agents

In an effort to identify new agents, a biological compound muramyl tripeptide phosphatidyl ethanolamine encapsulated in liposome (L-MTP-PE) was investigated. It was combined with chemotherapy in a 2×2 randomized factorial trial by the Children's Oncology Group [52]. It was administered after surgical resection of the primary tumor treated initially with neoadjuvant chemotherapy: cisplatin, doxorubicin, and high dose methotrexate. One-half of the patients were also randomly assigned to receive ifosfamide. In a second randomization they were assigned to receive L-MTP-PE after definitive surgical resection of the primary tumor. The addition of ifosfamide did not improve the outcome. The addition of the biological compound improved event free survival but did not meet the conventional test for statistical significance (*P* = 0.08) nor for a significant improvement in overall survival (78% versus 70%; *P* = 0.3). The role of L-MTP-PE in the United States remains under discussion; it is available by request on a compassionate Investigational New Drug (IND) application It has been accepted for use in Europe, but in the European ESMO guidelines no consensus could be reached on its use and more prospective research was advised before it could be generally accepted by the experts. L-MTP-PE was further addressed in subsequent communications [53]. Additional acceptance in other centers followed without alteration of its status in the United States.

## 6. Neoadjuvant Therapy 

Preoperative agents administered to treat the primary tumor to determine their potential use as postoperative treatment are designated “neoadjuvant therapy” a term introduced by Emil Frei III in discussing a presentation by Gerry Rosen at an American Society of Clinical Oncology (ASCO) meeting in the 1980s. Initially, the concept and rationale for administration of neoadjuvant chemotherapy were met with some resistance. However it appeared that the strategy could confer several local and systemic advantages: it could serve as an *in vivo/in vitro *trial for the selection of the postoperative agents as adjuvant therapy if a good response was obtained with the preoperative treatment; alternatively if ineffective, alternative agents would be introduced. Necrosis > 90% attained with preoperative chemotherapy is considered a good prognostic factor, whereas necrosis < 90% would be an indication for a possible change in the regimen.

Most studies currently advocate the deployment of neoadjuvant therapy. However several preliminary reports suggest that the results in long follow-up are similar in either circumstance. To address the controversy an international cooperative study, EURAMOS, has been formed to test the neoadjuvant hypothesis and other aspects of osteosarcoma [54]. The aim is to determine with greater confidence the potential for adding additional chemotherapeutic agents in order to improve outcome in patients whose tumors demonstrate a poor histological response to preoperative chemotherapy. In addition to that, the added value of interferon in good responders is being investigated. It is possible that the study may provide insight into biological and other variants which may impact response. This could provide information for construction of protocols for personalized treatment with chemotherapy. This is considered to be the new paradigm for treatment of the future.

Preoperative chemotherapy in EURAMOS comprises methotrexate, adriamycin, and cisplatin (MAP). Two different questions have then been posed for patients with either good or poor histological response: Favorable histological response (<10% viable tumor): patients receive the same agents administered preoperatively. They are also randomly assigned to receive additional therapy with pegylated interferon alpha-2b. Unfavorable histological response (10%–100%) viable tumor: patients randomly assigned to receive the same preoperative chemotherapy postoperatively plus or minus ifosfamide/etoposide.

## 7. Management of the Primary Tumor 

Optimum treatment for osteosarcoma demands a multidisciplinary strategy. While the effective and judicious application of chemotherapy has substantially changed the prognosis, it must be accompanied by appropriate local control to achieve cure. Surgical ablation of the diseased bone with oncologically safe margins is the best means of local control. For decades amputation and ablative surgery were widely practiced in an attempt to remove the tumor with safe margins and the least chance of local relapse. The advent of better imaging modalities, more effective chemotherapy, a better understanding of anatomy with continuous refinement in surgical techniques, and advances in prosthesis design and materials have all played a part in increasing the incidence of limb preserving surgery in osteosarcoma [55, 56]. From an era where amputation was the only option to the current day function preserving resections and complex reconstructions has been a major advance.

While the number of limb salvage surgeries undertaken for malignant bone tumors of the extremity has increased, the principles that govern surgical resection of bone tumors remain unchanged. The surgeon must ensure adequate resection of involved bone and soft tissue so as to minimize the chance of local recurrence. If after achieving this goal he is still able to preserve adequate function of the limb after reconstruction, then the patient is a suitable candidate for limb salvage. At no stage must adequate disease clearance be compromised in an attempt to achieve limb salvage.

Kawaguchi's concept of “barrier effects” helped surgeons better understand evaluation of margins of resection [57]. Though conventionally quantitative parameters were used to define resection margins Kawaguchi converted anatomical structures (any tissue that has resistance against tumor invasion like muscle fascia, joint capsule, tendon, tendon sheath, epineurium, vascular sheath, and cartilage) into definitive thickness of normal tissue and classified them as either a thick barrier or a thin barrier. For purposes of margin evaluation a thick barrier was equivalent of 3 cm thickness of normal tissue, a thin barrier was considered to be 2 cm, and joint cartilage 5 cm. By considering barrier effects translated into concrete distance equivalents, oncologically safe surgery can be planned at sites where barriers exist by using margins less than those mandated by true physical distance.

The advent of computer-assisted tumor surgery (CATS) in malignant bone tumors has increased the accuracy of intended bone resection and may be beneficial in resection and reconstruction of pelvic, sacral, and difficult joint-preserving tumor surgery [58]. It provides a useful tool in achieving a better balance between disease resection and preservation of function in anatomically challenging locations.

There are a variety of reconstruction options after excision of osteosarcoma. Metallic prostheses (megaprostheses) which span the resection gap and allow for movement of the joint form the mainstay in limb salvage surgery for reconstruction after tumor resection, providing both mobility and stability. Biological means of reconstruction using autografts, allografts, and reimplantation of sterilized tumor bone (after autoclaving/pasteurization/irradiation) offer an attractive alternative option in certain scenarios.

Though not the first choice for local control in these lesions, the advent of newer techniques of delivery has resulted in radiation playing an increasing role in unresectable lesions or after incomplete resection. Proton therapy and carbon ion radiotherapy have demonstrated acceptable local control and a survival advantage with acceptable morbidity in the management of unresectable or incompletely resected osteosarcoma [59, 60]. Use of other nonconventional modalities for local control like microwave induced hyperthermia and high intensity focused ultrasound have also shown promising results [61, 62]. These techniques may thus eventually have the potential to be utilized as one of the components of limb sparing options in patients with malignant bone tumors.

## 8. The Future

Despite the current impasse in an inability to improve survival, the future for patients afflicted with cancer appears to hold exciting possibilities for further advancement. Such advances will probably accrue with the introduction of personalized medical care based upon molecular diagnoses of individual tumors. Advances in diagnostic procedures particularly imaging studies will probably improve the ability for more accurate staging and possibly contribute to better identification of subtle metastases. Molecular diagnostic procedures and identification of tumors permitting more specific therapy are currently in use in several tumors and may possibly be extended to osteosarcoma in the forseeable future. Ultimately these advances are also predicated on the discovery of new chemotherapeutic agents and alternate mechanisms of therapy. 

## 9. Summary 

With the introduction of effective chemotherapeutic agents during the 1960s–1980s cure in osteosarcoma was escalated from <10% to 60%–75%. Approximately 80% of patients are currently considered eligible for limb salvage. While major advances have been achieved with chemotherapy, the results have been stagnant over the past thirty to forty years. New types of chemotherapy and new modes of treatment are urgently required. The EURAMOS study is currently designed to explore new avenues of investigation. It particularly includes an assessment of the utility of neoadjuvant treatment. Possibly the discovery of new biological variants and other factors may prove useful in designing personalized therapy for the future. Realistic new targets must be identified utilizing lessons from the past to achieve new levels of success.
